# Crystal structure of tetra­wickmanite, Mn^2+^Sn^4+^(OH)_6_

**DOI:** 10.1107/S2056989015001632

**Published:** 2015-01-31

**Authors:** Barbara Lafuente, Hexiong Yang, Robert T. Downs

**Affiliations:** a Department of Geosciences, University of Arizona, 1040 E. 4th Street, Tucson, AZ 85721-0077, USA

**Keywords:** crystal structure, tetra­wickmanite, mineral structure, polymorphism.

## Abstract

The crystal structure of tetra­wickmanite, ideally Mn^2+^Sn^4+^(OH)_6_ [mangan­ese(II) tin(IV) hexa­hydroxide], has been determined based on single-crystal X-ray diffraction data collected from a natural sample from Långban, Sweden. Tetra­wickmanite belongs to the octa­hedral-framework group of hydroxide-perovskite minerals, described by the general formula *BB*’(OH)_6_ with a perovskite derivative structure. The structure differs from that of an *AB*O_3_ perovskite in that the *A* site is empty while each O atom is bonded to an H atom. The perovskite *B*-type cations split into ordered *B* and *B*′ sites, which are occupied by Mn^2+^ and Sn^4+^, respectively. Tetra­wickmanite exhibits tetra­gonal symmetry and is topologically similar to its cubic polymorph, wickmanite. The tetra­wickmanite structure is characterized by a framework of alternating corner-linked [Mn^2+^(OH)_6_] and [Sn^4+^(OH)_6_] octa­hedra, both with point-group symmetry -1. Four of the five distinct H atoms in the structure are statistically disordered. The vacant *A* site is in a cavity in the centre of a distorted cube formed by eight octa­hedra at the corners. However, the hydrogen-atom positions and their hydrogen bonds are not equivalent in every cavity, resulting in two distinct environments. One of the cavities contains a ring of four hydrogen bonds, similar to that found in wickmanite, while the other cavity is more distorted and forms crankshaft-type chains of hydrogen bonds, as previously proposed for tetra­gonal stottite, Fe^2+^Ge^4+^(OH)_6_.

## Abstract 2

The crystal structure of tetra­wickmanite, a tetra­gonal hydroxide-perovskite mineral, has been determined for the first time by means of single-crystal X-ray diffraction. It is characterized by alternating corner-linked [Mn^2+^(OH)_6_] and [Sn^4+^(OH)_6_] octa­hedra whose sense of rotation varies along *c*, in contrast to its dimorph, the cubic wickmanite.

## Mineralogical and crystal-chemical context   

Tetra­wickmanite, ideally Mn^2+^Sn^4+^(OH)_6_, belongs to the octa­hedral-framework group of hydroxide-perovskites, described by the general formula *BB’*(OH)_6_ with a perovskite derivative structure. The structure of hydroxide-perovskites differs from that of an *AB*O_3_ perovskite in that the *A* site is empty while each O atom is bonded to a hydrogen atom. The lack of *A*-site cations makes them more compressible than perovskite structures (Kleppe *et al.*, 2012 ▸) and elicits an industrial inter­est for their potential use in hydrogen storage at high pressures (Welch & Wunder, 2012 ▸).

The hydroxide-perovskite species with *B* = *B′* include dzhalindite [In(OH)_3_] (Genkin & Murav’eva, 1963 ▸), bernalite [Fe^3+^(OH)_3_] (Birch *et al.*, 1993 ▸) and söhngeite [Ga(OH)_3_] (Strunz, 1965 ▸). The species with *B* ≠ *B′* have the two cations fully ordered into *B* and *B′* sites according to bond-valence constraints on the bridging O atoms. Valence states can range from +I to +III for *B*-site cations and from +III to +V for *B′*-site cations.

Tetra­wickmanite belongs to the group of hydrox­idostannate(IV) perovskites [*B*Sn^4+^(OH)_6_] which may exhibit cubic (*Pn*3, *Pn*3*m*) or tetra­gonal (*P*4_2_/*n*, *P*4_2_/*nnm*) symmetries. Burtite (*B* = Ca) (Sonnet, 1981 ▸), natanite (*B* = Fe^2+^) (Marshukova *et al.*, 1981 ▸), schoenfliesite (*B* = Mg) (Faust & Schaller, 1971 ▸), vismirnovite (*B* = Zn) (Marshukova *et al.*, 1981 ▸) and wickmanite (*B* = Mn^2+^) (Moore & Smith, 1967 ▸; Christensen & Hazell, 1969 ▸) display cubic symmetry while tetra­wickmanite (*B* = Mn^2+^), jeanbandyite (*B* = Fe^3+^) (Kampf, 1982 ▸) and mushistonite (*B* = Cu^2+^) (Marshukova *et al.*, 1984 ▸) are tetra­gonal. The two hydroxide-perovskites stottite (*B* = Fe^2+^, *B′* = Ge^4+^) (Strunz *et al.*, 1958 ▸) and mopungite (*B* = Na, *B′* = Sb^5+^) (Williams, 1985 ▸) are also tetra­gonal.

Tetra­wickmanite was initially described by White & Nelen (1973 ▸) from a pegmatite at the Foote Mineral Company’s spodumene mine, Kings Mountain, North Carolina. From the X-ray diffraction pattern and the crystal morphology, they determined that tetra­wickmanite exhibits tetra­gonal symmetry and is topologically similar to its polymorph, the cubic wickmanite. A second occurrence of tetra­wickmanite at Långban, Sweden, was reported by Dunn (1978 ▸) and described as tungsten-rich tetra­wickmanite with tungsten substituting for tin in the structure.

In the course of identifying minerals for the RRUFF Project (http://rruff.info), we were able to isolate a single crystal of tetra­wickmanite from Långban with composition (Mn^2+^_0.94_Mg_0.05_Fe^2+^_0.01_)_Σ=1_(Sn^4+^_0.92_W^6+^_0.05_)_Σ=0.97_(OH)_6_. Thereby, this study presents the first crystal structure determination of tetra­wickmanite by means of single-crystal X-ray diffraction.

## Structural commentary   

**Figure 1:**
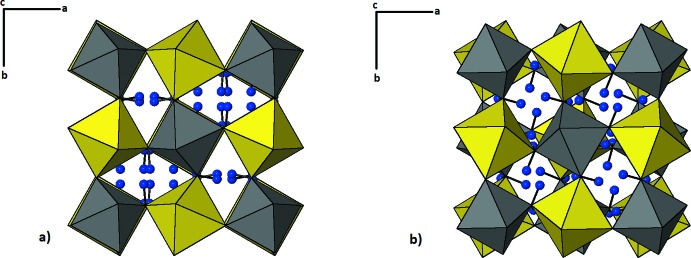
Framework of alternating corner-linked [Mn^2+^(OH)_6_] and [Sn^4+^(OH)_6_] octa­hedra in (*a*) wickmanite (Basciano *et al.*, 1998 ▸) and (*b*) tetra­wickmanite, with change in senses of rotation in alternate layers along the *c*-axis direction. Yellow and grey octa­hedra represent Mn and Sn sites, respectively. Blue spheres represent H atoms.

**Figure 2:**
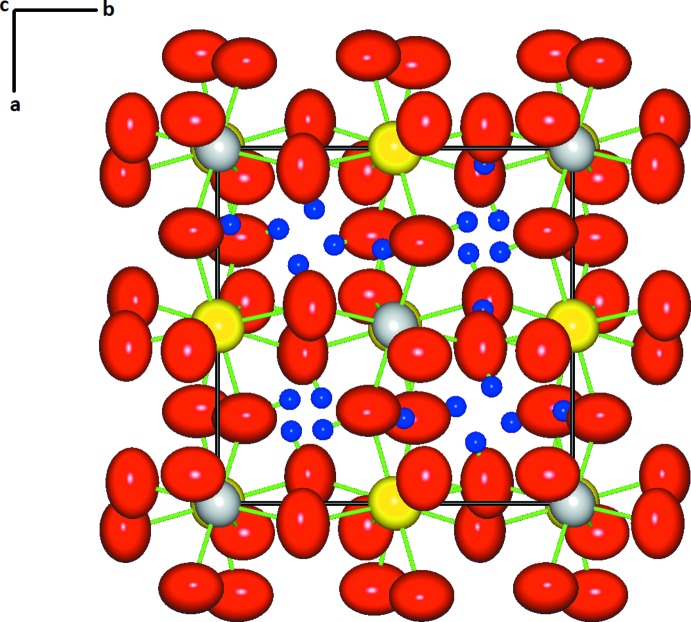
The crystal structure of tetra­wickmanite showing atoms with anisotropic displacement ellipsoids at the 99% probability level. Yellow, grey and red ellipsoids represent Mn, Sn and O atoms, respectively. Blue spheres of arbitrary radius represent H atoms.

The structure of tetra­wickmanite is characterized by a framework of alternating corner-linked [Mn^2+^(OH)_6_] and [Sn^4+^(OH)_6_] octa­hedra, centred at special positions 4*d* and 4*c*, respectively (site symmetry 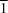) (Fig. 1 ▸ *b*). The Mn—O distances are 2.2007 (13), 2.1933 (12) and 2.2009 (14) Å (average 2.198 Å) and the Sn—O distances are 2.0654 (13), 2.0523 (12) and 2.0446 (13) Å (average 2.054 Å), both similar to the inter­atomic distances determined from neutron powder diffraction data for synthetic wickmanite (Mn—O average 2.181 Å and Sn—O average 2.055 Å; Basciano *et al.*, 1998 ▸). The tetra­wickmanite structure contains three non-equivalent O atoms, all protonated as OH groups and located at general positions. H1, H2, H3 and H4 are statistically disordered within the structure while H5 is ordered (Fig. 2 ▸).

Hydroxide-perovskites have the vacant *A* site in a cavity in the centre of a distorted cube formed by eight octa­hedra at the corners. According to the Glazer notation for octa­hedral-tilt systems in perovskites (Glazer, 1972 ▸), wickmanite, the cubic polymorph of tetra­wickmanite, is an *a* ^+^ *a* ^+^ *a* ^+^-type perovskite, with three equal rotations (Fig. 1 ▸ *a*) while tetra­wickmanite is of *a* ^+^ *a* ^+^ *c* ^−^ type and it changes the senses of rotation in alternate layers along the *c*-axis direction (Fig. 1 ▸ *b*). This difference in octa­hedral-tilt systems is similar to that observed during compressibility studies of cubic burtite [CaSn^4+^(OH)_6_; Welch & Crichton, 2002 ▸] and tetra­gonal stottite [Fe^2+^Ge^4+^(OH)_6_; Ross *et al.*, 2002 ▸]. As the authors pointed out, the variance in the octa­hedral-tilt systems leads to distinct hydrogen-bonding topologies between burtite and stottite, similar to those observed between wickmanite and tetra­wickmanite.

**Figure 3:**
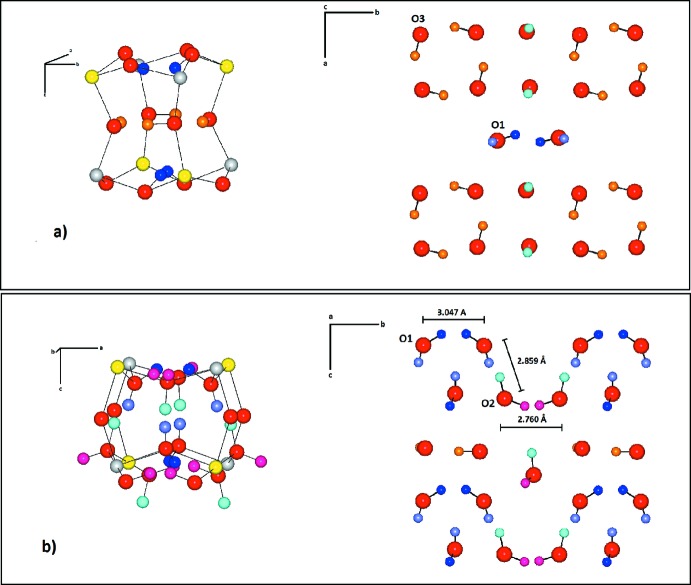
Cavity (left) and hydrogen-bonding linkages (right) in tetra­wickmanite. (*a*) Wickmanite-like cavity with isolated four-membered ring motif O3—H5⋯O3 and linkages O1—H1⋯O1 at the top and bottom of the cavity. (*b*) Sets of <100> crankshaft-type motifs with the isolated four-membered rings lining in the plane perpendicular to the *c* axis. Yellow, grey and red spheres represent Mn, Sn and O atoms. Blue, purple, pink, aqua­marine and orange spheres represent H1, H2, H3, H4 and H5 hydrogen atoms, respectively.

Wickmanite has a single type of cavity with the H atom disordered over two positions, forming a ring of four hydrogen-bonds with two other hydrogen-bonds at the top and the bottom of the cavity (Basciano *et al.*, 1998 ▸). However, in tetra­wickmanite, the hydrogen positions and their hydrogen bonds (Table 1 ▸) are not equivalent in every cavity, and exhibit two distinct environments. One of the cavities is similar to that of wickmanite, with isolated four-membered hydrogen-bonding ring motifs defined by O3—H5⋯O3 [2.752 (2) Å] and linkages O1—H1⋯O1 [3.047 (3) Å] at the top and bottom of the cavity (Fig. 3 ▸ *a*). In tetra­wickmanite, the four-membered ring has equal O3⋯O3 distances [2.752 (2) Å] while in wickmanite, the O⋯O distances alternate between 2.928 and 2.752 Å. Presumably, the shorter O⋯O distances within the ring motif in tetra­wickmanite is correlated with the ordering of the H5 atom.

The other cavity in tetra­wickmanite is more distorted, with the four-membered rings converted into <100> crankshaft-type motifs defined by three hydrogen bonds: O2—H3⋯O2 [2.760 (3) Å], O1—H2⋯O2/ O2—H4⋯O1 [2.859 (2) Å] and O1—H1⋯O1 [3.047 (3) Å] and the isolated four-membered rings lying in the plane perpendicular to the *c* axes. The hydrogen bonds O2—H3⋯O1 [3.140 (2) Å] and O1—H1⋯O2 [3.085 (2) Å] are located between the crankshafts, at the top and the bottom, respectively (Fig. 3 ▸ *b*). There are no hydrogen bonds parallel to [001].

As stated earlier, the compressibilities of cubic burtite and tetra­gonal stottite, with unit-cell volumes 535.8 and 426 Å^3^, respectively, have been studied and their hydrogen bonding has been compared (Welch & Crichton, 2002 ▸; Ross *et al.*, 2002 ▸). By analogy, a study of the compressibility of the polymorphs wickmanite and tetra­wickmanite, with much closer unit-cell volume values (488.26 and 482.17 Å^3^, respectively), might also help in understanding the connection between hydrogen-bonding topologies and compression mechanisms in hydroxide-perovskites.

Kleppe *et al.* (2012 ▸) studied pressure-induced phase trans­itions in hydroxide-perovskites based on Raman spectroscopy measurements of stottite [Fe^2+^Ge^4+^(OH)_6_] up to 21 GPa. In their work, they proposed the monoclinic space group *P*2/*n* for stottite at ambient conditions derived from the presence of six OH-stretching bands in the Raman spectra in the range 3064–3352 cm^−1^. We refined the structure of tetra­wickmanite in space group *P*2/*n* (*R*_1_ = 0.0215) and performed the Hamilton reliability test (Hamilton, 1965 ▸). The test indicated that the better structural model for tetra­wickmanite is based on the tetra­gonal space group *P*4_2_/*n* at the 92% confidence level. Moreover, analysis of the anisotropic displacement parameters showed that the tetra­gonal model displays ideal rigid-body motion of the strong polyhedral groups (Downs, 2000 ▸), thus corroborating a tetra­gonal structure for tetra­wickmanite.

**Figure 4:**
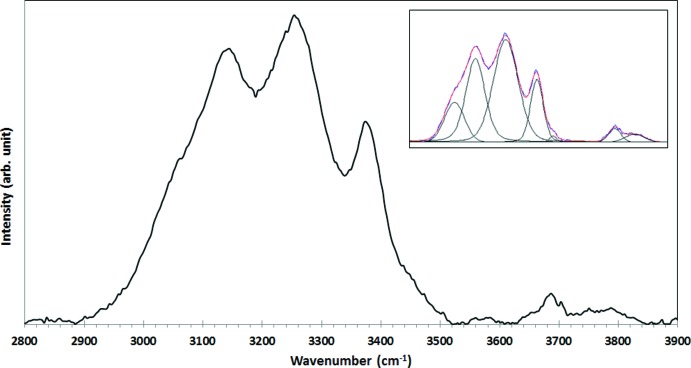
Raman spectrum of tetra­wickmanite in the OH-stretching region (2800–3900 cm^−1^). At the top right, the spectral deconvolution obtained with seven fitting peaks using pseudo-Voigt line profiles.

The Raman spectrum of tetra­wickmanite in the OH-stretching region (2800–3900 cm^−1^) is displayed in Fig. 4 ▸. The minimum number of peaks needed to fit the spectrum in this region (using pseudo-Voigt line profiles) is seven, which is in agreement with the number of hydrogen bonds derived from the structure (Table 1 ▸). According to the correlation of O—H stretching frequencies and O—H⋯O hydrogen-bond lengths in minerals by Libowitzky (1999 ▸), the most intense peaks (3062, 3145, 3253 and 3374 cm^−1^) are within the range of calculated wavenumbers for the H⋯O distances between 2.75 and 2.86 Å and they correspond to the strongest hydrogen bonds in the structure.

## Experimental   

The tetra­wickmanite specimen used in this study was from Långban, Sweden, and is in the collection of the RRUFF project (deposition R100003: http://rruff.info/R100003). Its chemical composition was determined with a CAMECA SX100 electron microprobe at the conditions of 20 kV, 20 nA and a beam size of 5 mm.

The analysis of thirteen points yielded an average composition (wt. %): MnO 24.47 (15), MgO 0.71 (11), FeO 0.34 (19), SnO_2_ 50.57 (15) and WO_3_ 4.49(1.21) with H_2_O 19.76 added to obtain a total close to 100%. The empirical chemical formula, calculated based on six oxygen atoms, is (Mn^2+^_0.94_Mg_0.05_Fe^2+^_0.01_)_Σ=1_(Sn^4+^_0.92_W^6+^_0.05_)_Σ=0.97_(OH)_6_.

The Raman spectrum of tetra­wickmanite was collected from a randomly oriented crystal on a Thermo-Almega microRaman system, using a 532 nm solid-state laser with a thermoelectric cooled CCD detector. The laser was partially polarized with 4 cm^−1^ resolution and a spot size of 1 mm.

## Refinement   

Crystal data, data collection and structure refinement details are summarized in Table 2 ▸. Electron microprobe analysis revealed that the tetra­wickmanite sample studied here contains small amounts of W, Mg and Fe. However, the structure refinements with and without a minor contribution of these elements in the octa­hedral sites did not produce any significant differences in terms of reliability factors or displacement parameters. Hence, the ideal chemical formula Mn^2+^Sn^4+^(OH)_6_ was assumed during the refinement, and all non-hydrogen atoms were refined with anisotropic displacement parameters. All H atoms were located from difference Fourier syntheses. The hydrogen atoms H1–H4 were modelled as statistically disordered around the parent O atom. H atom positions were refined freely; a fixed isotropic displacement parameter (*U*_iso_ = 0.03 Å) was used for all H atoms.

The maximum residual electron density in the difference Fourier map, 0.55 e Å^−3^, was located at (0.7590 0.5372 0.0856), 1.28 Å from H5 and the minimum, −0.54 e Å^−3^, at (0.7181 0.5102 0.2313), 0.22 Å from H5.

## Supplementary Material

Crystal structure: contains datablock(s) I. DOI: 10.1107/S2056989015001632/wm5112sup1.cif

Structure factors: contains datablock(s) I. DOI: 10.1107/S2056989015001632/wm5112Isup2.hkl

CCDC reference: 1045459

Additional supporting information:  crystallographic information; 3D view; checkCIF report

## Figures and Tables

**Table 1:** Hydrogen-bond geometry (, )

*D*H*A*	*D*H	H*A*	*D* *A*	*D*H*A*
O1H1O1^i^	1.10(6)	2.22(7)	3.047(3)	131(4)
O1H1O2^i^	1.10(6)	2.51(6)	3.0846(19)	111(4)
O1H2O2^ii^	0.89(7)	1.98(7)	2.859(2)	171(5)
O2H3O2^iii^	1.15(7)	1.80(7)	2.760(3)	138(3)
O2H3O1^iv^	1.15(7)	2.30(5)	3.140(2)	128(4)
O2H4O1^v^	1.11(5)	1.77(5)	2.859(2)	165(5)
O3H5O3^vi^	1.09(3)	1.74(3)	2.752(2)	153(3)

Symmetry codes: (i) 
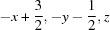
; (ii) 
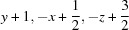
; (iii) 
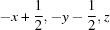
; (iv) 
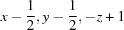
; (v) 
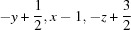
; (vi) 
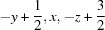
.

**Table 2:** Experimental details

Crystal data	
Chemical formula	MnSn(OH)_6_
*M*_r_	275.68
Crystal system, space group	Tetragonal, *P*4_2_/*n*
Temperature (K)	293
*a*, *c* ()	7.8655(4), 7.7938(6)
*V* (^3^)	482.17(5)
*Z*	4
Radiation type	Mo *K*
(mm^1^)	7.74
Crystal size (mm)	0.05 0.05 0.04

Data collection	
Diffractometer	Bruker APEXII CCD area detector
Absorption correction	Multi-scan (*SADABS*; Bruker, 2004 ▸)
*T*_min_, *T*_max_	0.698, 0.747
No. of measured, independent and observed [*I* > 2(*I*)] reflections	4394, 1272, 681
*R*_int_	0.020
(sin /)_max_ (^1^)	0.863

Refinement	
*R*[*F* ^2^ > 2(*F* ^2^)], *wR*(*F* ^2^), *S*	0.021, 0.056, 1.00
No. of reflections	1272
No. of parameters	56
H-atom treatment	All H-atom parameters refined
_max_, _min_ (e ^3^)	0.55, 0.54

Computer programs: *APEX2* and *SAINT* (Bruker, 2004 ▸), *SHELXS97* and *SHELXL97* (Sheldrick, 2008 ▸), *XtalDraw* (Downs Hall-Wallace, 2003 ▸) and *publCIF* (Westrip, 2010 ▸).

## supplementary crystallographic information

### Crystal data

**Table:**

MnSn(OH)_6_	*D*_x_ = 3.798 Mg m^−^^3^
*M**_r_* = 275.68	Mo *K*α radiation, λ = 0.71073 Å
Tetragonal, *P*4_2_/*n*	Cell parameters from 1249 reflections
Hall symbol: -P 4bc	θ = 4.5–37.8°
*a* = 7.8655 (4) Å	µ = 7.74 mm^−^^1^
*c* = 7.7938 (6) Å	*T* = 293 K
*V* = 482.17 (5) Å^3^	Pseudocubic, yellow–orange
*Z* = 4	0.05 × 0.05 × 0.04 mm
*F*(000) = 516	

### Data collection

**Table:**

Bruker APEXII CCD area-detector diffractometer	1272 independent reflections
Radiation source: fine-focus sealed tube	681 reflections with *I* > 2σ(*I*)
Graphite monochromator	*R*_int_ = 0.020
φ and ω scan	θ_max_ = 37.8°, θ_min_ = 3.7°
Absorption correction: multi-scan (*SADABS*; Bruker, 2004)	*h* = −11→7
*T*_min_ = 0.698, *T*_max_ = 0.747	*k* = −12→13
4394 measured reflections	*l* = −13→5

### Refinement

**Table:**

Refinement on *F*^2^	Secondary atom site location: difference Fourier map
Least-squares matrix: full	Hydrogen site location: difference Fourier map
*R*[*F*^2^ > 2σ(*F*^2^)] = 0.021	All H-atom parameters refined
*wR*(*F*^2^) = 0.056	*w* = 1/[σ^2^(*F*_o_^2^) + (0.022*P*)^2^] where *P* = (*F*_o_^2^ + 2*F*_c_^2^)/3
*S* = 1.00	(Δ/σ)_max_ < 0.001
1272 reflections	Δρ_max_ = 0.55 e Å^−^^3^
56 parameters	Δρ_min_ = −0.54 e Å^−^^3^
0 restraints	Extinction correction: *SHELXL97* (Sheldrick, 2008), Fc^*^=kFc[1+0.001xFc^2^λ^3^/sin(2θ)]^-1/4^
Primary atom site location: structure-invariant direct methods	Extinction coefficient: 0.0044 (3)

### Special details

**Table:**

Geometry. All e.s.d.'s (except the e.s.d. in the dihedral angle between two l.s. planes) are estimated using the full covariance matrix. The cell e.s.d.'s are taken into account individually in the estimation of e.s.d.'s in distances, angles and torsion angles; correlations between e.s.d.'s in cell parameters are only used when they are defined by crystal symmetry. An approximate (isotropic) treatment of cell e.s.d.'s is used for estimating e.s.d.'s involving l.s. planes.
Refinement. Refinement of *F*^2^ against ALL reflections. The weighted *R*-factor *wR* and goodness of fit *S* are based on *F*^2^, conventional *R*-factors *R* are based on *F*, with *F* set to zero for negative *F*^2^. The threshold expression of *F*^2^ > σ(*F*^2^) is used only for calculating *R*-factors(gt) *etc*. and is not relevant to the choice of reflections for refinement. *R*-factors based on *F*^2^ are statistically about twice as large as those based on *F*, and *R*- factors based on ALL data will be even larger.

### Fractional atomic coordinates and isotropic or equivalent isotropic displacement parameters (Å^2^)

**Table:**

	*x*	*y*	*z*	*U*_iso_*/*U*_eq_	Occ. (<1)
Sn	0.5000	0.0000	0.5000	0.00790 (6)	
Mn	0.5000	0.0000	0.0000	0.01017 (8)	
O1	0.74065 (17)	−0.05652 (19)	0.5894 (2)	0.0133 (3)	
O2	0.42512 (19)	−0.23929 (17)	0.56923 (18)	0.0138 (3)	
O3	0.43079 (18)	0.08165 (18)	0.74014 (15)	0.0113 (3)	
H1	0.772 (7)	−0.171 (8)	0.517 (7)	0.030*	0.50
H2	0.740 (7)	−0.025 (7)	0.699 (9)	0.030*	0.50
H3	0.297 (9)	−0.292 (7)	0.525 (4)	0.030*	0.50
H4	0.455 (8)	−0.252 (7)	0.707 (7)	0.030*	0.50
H5	0.465 (4)	0.215 (4)	0.743 (2)	0.030*	

### Atomic displacement parameters (Å^2^)

**Table:**

	*U*^11^	*U*^22^	*U*^33^	*U*^12^	*U*^13^	*U*^23^
Sn	0.00751 (11)	0.00801 (11)	0.00817 (8)	−0.00042 (8)	0.00044 (6)	0.00029 (6)
Mn	0.0102 (2)	0.0102 (2)	0.01012 (17)	0.0004 (2)	0.00047 (14)	0.00021 (15)
O1	0.0098 (6)	0.0174 (7)	0.0128 (6)	−0.0002 (5)	−0.0011 (6)	0.0014 (6)
O2	0.0147 (7)	0.0091 (7)	0.0176 (7)	0.0001 (5)	0.0009 (6)	0.0012 (6)
O3	0.0145 (7)	0.0107 (8)	0.0087 (5)	−0.0002 (6)	0.0005 (5)	−0.0006 (5)

### Geometric parameters (Å, º)

**Table:**

Sn—O2^i^	2.0446 (13)	Mn—O3^ii^	2.1933 (12)
Sn—O2	2.0446 (13)	Mn—O3^i^	2.1933 (12)
Sn—O3	2.0523 (12)	Mn—O2^iii^	2.2007 (13)
Sn—O3^i^	2.0523 (12)	Mn—O2^iv^	2.2007 (13)
Sn—O1^i^	2.0654 (13)	Mn—O1^v^	2.2009 (14)
Sn—O1	2.0654 (13)	Mn—O1^vi^	2.2009 (14)
			
O2^i^—Sn—O2	180.0	O3^ii^—Mn—O3^i^	180.00 (7)
O2^i^—Sn—O3	91.66 (5)	O3^ii^—Mn—O2^iii^	94.21 (5)
O2—Sn—O3	88.34 (5)	O3^i^—Mn—O2^iii^	85.79 (5)
O2^i^—Sn—O3^i^	88.34 (5)	O3^ii^—Mn—O2^iv^	85.79 (5)
O2—Sn—O3^i^	91.66 (5)	O3^i^—Mn—O2^iv^	94.21 (5)
O3—Sn—O3^i^	180.00 (3)	O2^iii^—Mn—O2^iv^	180.00 (7)
O2^i^—Sn—O1^i^	88.67 (5)	O3^ii^—Mn—O1^v^	88.32 (5)
O2—Sn—O1^i^	91.33 (5)	O3^i^—Mn—O1^v^	91.68 (5)
O3—Sn—O1^i^	89.84 (6)	O2^iii^—Mn—O1^v^	88.98 (5)
O3^i^—Sn—O1^i^	90.16 (6)	O2^iv^—Mn—O1^v^	91.02 (5)
O2^i^—Sn—O1	91.33 (5)	O3^ii^—Mn—O1^vi^	91.68 (5)
O2—Sn—O1	88.67 (5)	O3^i^—Mn—O1^vi^	88.32 (5)
O3—Sn—O1	90.16 (6)	O2^iii^—Mn—O1^vi^	91.02 (5)
O3^i^—Sn—O1	89.84 (6)	O2^iv^—Mn—O1^vi^	88.98 (5)
O1^i^—Sn—O1	180.0	O1^v^—Mn—O1^vi^	180.0

Symmetry codes: (i) −*x*+1, −*y*, −*z*+1; (ii) *x*, *y*, *z*−1; (iii) −*y*, *x*−1/2, *z*−1/2; (iv) *y*+1, −*x*+1/2, −*z*+1/2; (v) *y*+1/2, −*x*+1, *z*−1/2; (vi) −*y*+1/2, *x*−1, −*z*+1/2.

### Hydrogen-bond geometry (Å, º)

**Table:**

*D*—H···*A*	*D*—H	H···*A*	*D*···*A*	*D*—H···*A*
O1—H1···O1^vii^	1.10 (6)	2.22 (7)	3.047 (3)	131 (4)
O1—H1···O2^vii^	1.10 (6)	2.51 (6)	3.0846 (19)	111 (4)
O1—H2···O2^viii^	0.89 (7)	1.98 (7)	2.859 (2)	171 (5)
O2—H3···O2^ix^	1.15 (7)	1.80 (7)	2.760 (3)	138 (3)
O2—H3···O1^x^	1.15 (7)	2.30 (5)	3.140 (2)	128 (4)
O2—H4···O1^xi^	1.11 (5)	1.77 (5)	2.859 (2)	165 (5)
O3—H5···O3^xii^	1.09 (3)	1.74 (3)	2.752 (2)	153 (3)

Symmetry codes: (vii) −*x*+3/2, −*y*−1/2, *z*; (viii) *y*+1, −*x*+1/2, −*z*+3/2; (ix) −*x*+1/2, −*y*−1/2, *z*; (x) *x*−1/2, *y*−1/2, −*z*+1; (xi) −*y*+1/2, *x*−1, −*z*+3/2; (xii) −*y*+1/2, *x*, −*z*+3/2.

## References

[bb1] Basciano, L. C., Peterson, R. C. & Roeder, P. L. (1998). *Can. Mineral.* **36**, 1203–1210.

[bb2] Birch, W. D., Pring, A., Reller, A. & Schmalle, H. D. (1993). *Am. Mineral.* **78**, 827–834.

[bb3] Bruker (2004). *APEX2*, *SAINT* and *SADABS*. Bruker AXS Inc., Madison, Wisconsin, USA.

[bb4] Christensen, A. N. & Hazell, R. G. (1969). *Acta Chem. Scand* **23**, 1219–1224.

[bb5] Downs, R. T. (2000). *Rev. Mineral. Geochem.* **41**, 61–88.

[bb6] Downs, R. T. & Hall-Wallace, M. (2003). *Am. Mineral.* **88**, 247–250.

[bb7] Dunn, P. J. (1978). *Miner. Rec.* **9**, 41–41.

[bb8] Faust, G. T. & Schaller, W. T. (1971). *Z. Kristallogr.* **134**, 116–141.

[bb9] Genkin, A. D. & Murav’eva, I. V. (1963). *Zap. Vses. Miner. Ob.* **92**, 445–457.

[bb10] Glazer, A. M. (1972). *Acta Cryst.* B**28**, 3384–3392.

[bb11] Hamilton, W. C. (1965). *Acta Cryst.* **18**, 502–510.

[bb12] Kampf, A. R. (1982). *Mineral. Rec* **13**, 235–239.

[bb13] Kleppe, A. K., Welch, M. D., Crichton, W. A. & Jephcoat, A. P. (2012). *Mineral. Mag.* **76**, 949–962.

[bb14] Libowitzky, E. (1999). *Monatsh. Chem.* **130**, 1047–1059.

[bb15] Marshukova, N. K., Palovskii, A. B. & Sidorenko, G. A. (1984). *Zap. Vses. Miner. Ob.* **113**, 612–617.

[bb16] Marshukova, N. K., Palovskii, A. B., Sidorenko, G. A. & Chistyakova, N. I. (1981). *Zap. Vses. Miner. Ob.* **110**, 492–500.

[bb17] Moore, P. B. & Smith, J. V. (1967). *Ark. Miner. Geol.* **4**, 395–399.

[bb18] Ross, N. L., Chaplin, T. D. & Welch, M. D. (2002). *Am. Mineral.* **87**, 1410–1414.

[bb19] Sheldrick, G. M. (2008). *Acta Cryst.* A**64**, 112–122. 10.1107/S0108767307043930 18156677

[bb20] Sonnet, P. M. (1981). *Can. Mineral.* **19**, 397–401.

[bb21] Strunz, H. (1965). *Naturwissenschaften*, **52**, 493–493.

[bb22] Strunz, H., Söhnge, G. & Geier, B. H. (1958). *Neues Jb. Miner. Mh.* **1958**, 85–96.

[bb23] Welch, M. D. & Crichton, W. A. (2002). *Miner. Mag.* **66**, 431–440.

[bb24] Welch, M. D. & Wunder, B. (2012). *Phys. Chem. Miner.* **39**, 693–697.

[bb25] Westrip, S. P. (2010). *J. Appl. Cryst.* **43**, 920–925.

[bb26] White, J. S. Jr & Nelen, J. A. (1973). *Miner. Rec.* **4**, 24–30.

[bb27] Williams, S. A. (1985). *Miner. Rec.* **16**, 73–74.

